# Cross-cultural adaptation and validation of the Slovenian version of the Core outcome measures index for low back pain

**DOI:** 10.1186/s12891-020-03280-8

**Published:** 2020-04-13

**Authors:** Matevž Topolovec, David Vozlič, Nejc Plohl, Rok Vengust, Miha Vodičar, Anne Frances Mannion

**Affiliations:** 1 Spine department, Orthopaedic hospital Valdoltra, Jadranska c.31, 6280 Ankaran, Slovenia; 2 Spinal surgery division, Department of Orthopaedic Surgery of the Ljubljana University Medical Centre, Zaloška cesta 9, 1000 Ljubljana, Slovenia; 3 Department of maxillofacial Surgery of the Ljubljana University Medical Centre, Zaloška cesta 2, 1000 Ljubljana, Slovenia; 4 Department of Psychology, Faculty of Arts, University of Maribor, Koroška cesta 160, 2000 Maribor, Slovenia; 5 Department of Teaching, Research and Development, Spine Center Division, Schulthess Klinik, Lengghalde 2, 8008 Zürich, Switzerland

**Keywords:** Cross-cultural adaptation, Validation, Core outcome measures index, Low back pain, Slovenian

## Abstract

**Background:**

To conduct a cross-cultural adaptation and validation of the Core Outcome Measures Index (COMI) in the Slovenian language, for use in patients with low back pain.

**Methods:**

The English version of COMI was translated into Slovene following established guidelines. Three hundred fifty-three patients with chronic low back pain were recruited from the Orthopedic clinic department of a tertiary care teaching institution. Data quality, construct validity, responsiveness, and test-retest reliability of the COMI were assessed.

**Results:**

The questionnaire was generally well accepted with no missing values. The majority of items exhibited only mild ceiling effects (below 20.0%) and somewhat more prominent floor effects, which were similar to previous studies (4.5–78.8%). Correlations with Oswestry Disability Index (ODI) were high (ρ = 0.76 between overall COMI and ODI scores), suggesting that the Slovene version of COMI had high construct validity. Additionally, the Slovene version of COMI successfully captured surgical patients’ improvement in their low back problem after surgery (overall COMI score change: Z = − 9.34, *p* < .001, r = − 0.53) and showed acceptable test-retest reliability (ICC = 0.86).

**Conclusions:**

The Slovene version of COMI showed good psychometric properties, comparable to those of previously tested language versions. It represents a valuable instrument for the use in future domestic and multicenter clinical studies.

## Background

Low back pain is one of the most common health problems and has a profound effect on both a personal and societal level. An adequate appraisal of the back problem and its consequences for the patient is essential when evaluating the effectiveness of different therapeutic approaches. The standardized measurement of outcome can facilitate scientific advances in clinical care [1].

Various low back disability scales have been proposed, including Short Form (SF)-36 [2], Quebec Back Pain Disability Scale [3], Oswestry Disability Index [4, 5] and others. However, these questionnaires are limited by their only assessing one domain. Most questionnaires are a compromise between survey length and precision [6, 7]. The Core Outcome Measures Index (COMI) [8, 9] is a brief, multidimensional instrument that has proven to be reliable, valid and highly responsive [9, 10] and has become the main tool for the Spine Tango registry of EUROSPINE [11]. It can be reliably used in clinical and research settings [6, 7]. Furthermore, it has been cross-culturally adapted and validated for use in many different languages (https://www.eurospine.org/forms.htm).

The aim of this study was to cross-culturally adapt the COMI for use in Slovene speaking patients and to assess the validity and responsiveness of the translated version.

## Methods

### The COMI

The Core Outcome Measures Index (COMI) is a short, multidimensional instrument that has one question each on back pain intensity, leg/buttock pain intensity, function, symptom-specific well-being, general quality of life, work disability and social disability, scored as a 0–10 index [8]. The COMI has been described in detail by Mannion et al. [8].

### Translation and cross-cultural adaptation

The translation and cross-cultural adaptation of the original English version of the COMI into Slovene was carried out in accordance with previously published guidelines [12].

Two bilingual translators whose first language was Slovene independently translated the original English version of the COMI to Slovene. The first translator (T1) was an expert in the field (Resident of Orthopedic surgery). The second translator (T2) was a High school teacher of English, not familiar with the concepts and the clinical content of the questionnaires. Both translators compared and discussed their versions and a consensus version (common Slovene translation T-12) was produced.

Back translation of T-12 into English was performed independently by native English speakers who were also fluent in the Slovene language. Both back-translators were blind to the original English version and had no medical knowledge. They were both working in a high school as assistant teachers.

A committee was formed consisting of one of the translators, one of the back translators, three spine surgeons and one methodologist research scientist. The committee examined all the translations and reached a consensus of the pre-final Slovene version. All stages of the translation process were documented in written form.

Fifteen surgical patients with chronic LBP were asked to fill out the pre-final version of COMI. After completing the questionnaire, they were asked about the content and the structure of it. The findings were then discussed and a final Slovene version was produced accordingly.

The study was approved by our Institutional Ethical Review Board. After giving their written informed consent, the patients received a booklet of questionnaires including items on demographic variables, the final Slovene COMI and the Oswestry Disability Index (ODI).

### Patients

The sample consisted of 353 patients from our Orthopedic clinic department who were administered the COMI questionnaire between January 2017 and March 2019. All patients indicated that they had problems with back pain, leg/buttock pain, or sensory disturbances in the back/leg/buttocks (e.g. tingling). Both sexes were relatively equally represented in the sample (47.0% male, 53.0% female) and the average age was 65.1 years (*SD* = 12.5; range: 25–87). Overall, 129 (36.5%) patients indicated that back pain was the problem that troubled them the most, 116 (32.9%) leg/buttock pain, and 108 (30.6%) sensory disturbances.

**Fig. 1:**
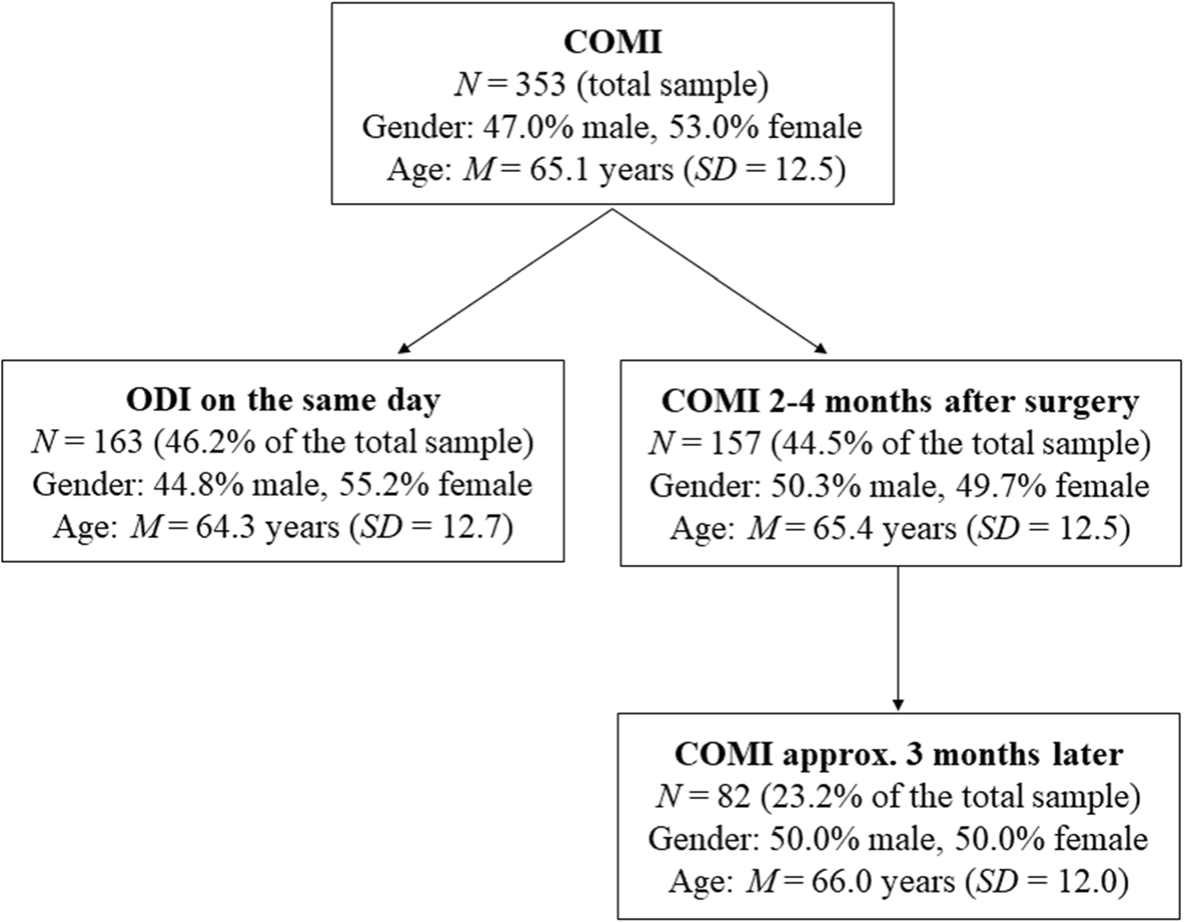
Flow diagram. Legend: ODI: Oswestry Disability Index; COMI: Core Outcome Measures Index; SD: standard deviation

Some analyses were performed on subsamples with similar basic demographic characteristics (gender, age, and the chief complaint) to the ones presented above. One part of the construct validity analysis was performed on a subsample of patients who had also filled out the Oswestry Disability Questionnaire on the same day (before surgery). The responsiveness part of the analysis was conducted on a subsample of participants who filled out the questionnaire again approximately 2–4 months after surgery (*M* = 81.1 days, *SD* = 13.6, range: 60–115 days after surgery). Additionally, the reliability (stability) part of the analysis was performed on a subsample of patients who filled out the questionnaire once more, approximately 3 months later (*M* = 81.8 days, *SD* = 5.6, range: 60–90 days after). All the subsamples are depicted in Fig. 1 below.

### Statistical analysis

The overall COMI score was computed as previously described [13]. It can range from 0 (best health status) to 10 (worst health status). Scores for Oswestry Disability Index were calculated as described by Fairbank and Pynsent [4], and ranged from 0 to 100, with higher scores indicating a higher severity of disability.

Missing data were analyzed for each COMI item and the overall score; specifically, we divided the number of missing values by the number of respondents in the sample. Floor and ceiling effects were assessed by calculating the percentage of respondents who, respectively, exhibited maximum and minimum possible scores on individual COMI items and the overall score. Floor and ceiling effects can make it impossible to detect deterioration or improvement in the participants’ status (e.g. if the value already indicates the best possible status, improvement cannot be detected) [14]. When interpreting these values, floor and ceiling effects larger than 70% are often considered to be adverse and effects smaller than 15% are often considered to be ideal [14, 15].

We also performed statistical analyses aimed at investigating construct validity, which refers to the degree to which scores on one instrument relate to other measures in a manner that is consistent with theoretically derived hypotheses [15, 16]. In other words, two questionnaires that measure the same construct or highly similar constructs, are expected to be (strongly) positively correlated. In the present study, these analyses were done by testing the relationship between individual COMI items, the overall COMI score, and a previously established instrument – the Oswestry Disability Index. Spearman Rho (ρ) corrected for ties was used in correlational analyses, and the following thresholds were used to interpret the calculated validity coefficients: ρ > .80 as excellent, .61–.80 very good, .41–.60 good, .21–.40 fair, and .00–.20 poor [17]. Based on previous studies that examined the relationship between ODI and COMI, we expect fair to good correlations between individual COMI items and the overall ODI score. Additionally, we expect to find very good to excellent correlation between the overall COMI and ODI scores [7, 18].

Responsiveness, one of the key attributes that needs to be considered when evaluating new questionnaires, is defined as the ability of a questionnaire to detect clinically important changes over time, even if these changes are small. A large number of methods have been proposed for assessing responsiveness [16]. In the present study, we used approaches that have already been used in previous COMI validation studies [18]. The change in group median scores from pre-surgery (baseline) to 3 months post-surgery were calculated using the Wilcoxon Signed Ranks Test, the non-parametric equivalent of the Paired Samples *T*-Test. We also calculated effect sizes (*r*) for the change scores [19], with values of 0.1 indicating small effects, 0.3 medium effects, and 0.5 large effects [8, 9, 18]. Additionally, we further explored the change in median scores based on the “global treatment outcome” question (i.e. Overall, how much did the operation help your back problem?). In other words, we aimed to find out whether the median change in the overall COMI score differed between patients who perceived the therapeutic intervention as being helpful or very helpful and those who perceived it as less efficacious [8, 9]. To compare median changes, we performed a non-parametric Mann-Whitney U test.

Lastly, we performed test-retest analyses, which explore questionnaires’ stability over time, by comparing COMI results at time-point 2 (2–4 months after surgery) with COMI results at time point 3 (approximately 3 months after time-point 2; see Fig. 1). A common method to evaluate this form of reliability is by calculating intraclass correlation coefficients (ICC) and their 95% confidence intervals. Intraclass correlation coefficients can occupy values between 0.0 and 1.0, with values of 0.6–0.8 generally indicating good reliability and values above 0.8 indicating excellent test-retest reliability [20]. While several previous COMI validations [7] show that COMI is a very reliable measure, it is worth noting that the time lag between the two COMI applications (with no therapeutic intervention in between) is a bit longer in our study (approximately 3 months as opposed to 2 weeks). As such, we expected slightly lower, but still satisfactory, test-retest values for each individual item as well as the overall COMI score. Standard errors of measurement (SEM) were also calculated and, in the next step, used to obtain data regarding the minimum detectable change (MDC95%) – the degree of change required in a patient’s score in order to establish it as being a real change, over and above measurement error. At the 95% confidence level, this is defined as 1.96 × √2 × SEM, which is equivalent to 2.77 × SEM.

All statistical analyses were performed with IBM SPSS 23.0 software; *p* values of less than .050 were considered significant.

## Results

### Score distribution, missing data

According to the Kolmogorov-Smirnov test, all of the individual COMI items, as well as the overall COMI score, violated the normality assumption (this is true in the case of the general sample and all of the subsamples; *p* < .001). This was taken into account in all of the following analyses; specifically, we used non-parametric tests, such as Spearman’s rho, Wilcoxon Signed Ranks test, and Mann-Whitney U test that do not assume a normal distribution of scores. We encountered no missing values when analyzing the COMI items (0.0%). In the same sample, however, we encountered missing data for the ODI questionnaire, with the item with the greatest % missing values being the “sex life” item (35.1% did not answer this question).

### Floor and ceiling effects

Table 1 also contains data on floor (i.e. the worst possible status) and ceiling effects (i.e. the best possible status) of each individual item and the overall COMI score. For the majority of items, we found extremely low ceiling effects (0–5.9%), while the “leg pain” item (13.0%) and the “work disability” item (46.2%) exhibited somewhat higher but still rather mild ceiling effects. The floor effect was a bit more pronounced, with just two individual items (back pain and leg pain) and the overall COMI score exhibiting extremely low floor effects (0–7.1%), four items exhibiting a higher floor effect (28.3–40.8%), and the “symptom-specific well-being” item having the most prominent floor effect (78.8%).

**Table 1:** Missing data, floor and ceiling effects

Core items (scoring)	Median (*IQR*)	Ceiling effect: best status (%)	Floor effect: worst status (%)
Back pain (0–10)	7.0 (3.0)	4.8	7.1
Leg pain (0–10)	7.0 (3.0)	13.0	6.5
Function (1–5)	4.0 (1.0)	0.8	38.5
Symptom-specific well-being (1–5)	5.0 (0.0)	0.3	78.8
Quality of life (1–5)	4.0 (2.0)	0.0	28.3
Social disability (1–5)	4.0 (2.0)	5.9	40.8
Work disability (1–5)	2.0 (4.0)	46.2	30.0
Overall COMI score (0–10)	7.7 (2.4)	0.0	4.5

*COMI* Core Outcome Measures Index; *IQR* Interquartile range

### Construct validity

The Spearman’s Rho correlations between the COMI items/summary score and the Oswestry Disability Index (ODI) are shown in Table 2. A very good correlation was found between the overall COMI score and the ODI, while the correlation coefficients between individual items and the ODI score can generally be summed up as fair to very good.

**Table 2:** Relationship between COMI items and ODI

Core items (scoring)	ODI score: correlation coefficient (Slovene adaptation)	ODI score: correlation coefficient (Polish adaptation [7])
Back pain (0–10)	0.65	0.30
Leg pain (0–10)	0.53	0.47
Function (1–5)	0.67	0.58
Symptom-specific well-being (1–5)	0.47	0.43
Quality of life (1–5)	0.65	0.52
Social disability (1–5)	0.55	0.49
Work disability (1–5)	0.35	0.49
Overall COMI score (0–10)	0.76	0.62

*ODI* Oswestry Disability Index; *COMI* Core Outcome Measures Index; All values are significant at *p* < 0.01

### Responsiveness

Table 3 shows the results of the responsiveness analysis. Surgical patients showed a significant improvement in the median scores of all the COMI items as well as the overall COMI score, which, on average, was 2.5 points lower, approximately 3 months after surgery. The effect size pertaining to the overall COMI score was large. Similar effect sizes were also seen in the case of many individual COMI items and the worst pain score; the most notable exception was for the “work disability” item, which exhibited a much smaller effect size.

**Table 3:** Results of the responsiveness analysis

	Baseline median (*IQR*)	Post-surgery median (*IQR*)	Wilcoxon Signed Ranks Test	Effect size (*r*)
Back pain (0–10)	7.0 (3.0)	4.0 (4.0)	*Z =* −8.15, *p* < .001	−0.45
Leg pain (0–10)	7.0 (3.0)	4.0 (5.0)	*Z* = −7.89, *p* < .001	− 0.45
Worst pain (0–10)	8.0 (2.0)	5.0 (4.0)	*Z* = −9.27, *p <* .001	− 0.52
Function (1–5)	4.0 (1.0)	3.0 (2.0)	*Z* = − 8.61, *p* < .001	− 0.49
Symptom-specific well-being (1–5)	5.0 (0.0)	4.0 (2.0)	*Z* = − 7.08, *p* < .001	− 0.40
Quality of life (1–5)	4.0 (2.0)	3.0 (2.0)	*Z* = − 8.29, *p* < .001	−0.47
Social disability (1–5)	4.0 (2.0)	3.0 (2.0)	*Z* = −5.88, *p* < .001	−0.33
Work disability (1–5)	2.0 (4.0)	2.0 (3.0)	*Z* = − 2.10, *p* = .039	−0.12
Overall COMI score (0–10)	7.8 (2.6)	5.3 (3.6)	*Z* = − 9.34, *p* < .001	−0.53

*COMI* Core Outcome Measures Index; *IQR* Interquartile range

Overall, 38 patients (24.2%) claimed that operation helped their back problem a lot, 81 (51.6%) that it “helped”, 29 (18.5%) that it “helped only little”, 7 (4.5%) that it “didn’t help”, and 2 (1.3%) that it “made things worse”. These patients were divided into two groups: a good outcome group (those who answered “helped a lot” or “helped”; *N* = 119) and a poor outcome group (all remaining patients; *N* = 38). Table 4 shows the change-scores for the individual COMI items and the overall COMI score for these two groups.

**Table 4:** Results of the responsiveness analysis: a comparison of two groups

	Good outcome group			Poor outcome group			
	Baseline median (*IQR*)	Post-surgery median (*IQR*)	Median change (*IQR*)	Baseline median (*IQR*)	Post-surgery median (*IQR*)	Median change (*IQR*)	Mann-Whitney U test
Back pain (0–10)	7.0 (3.0)	3.0 (3.0)	− 3.0 (4.0)	7.0 (3.0)	6.0 (3.0)	− 1.0 (2.0)	*Z* = − 3.26, *p* = .001
Leg pain (0–10)	7.0 (3.0)	3.0 (5.0)	−3.0 (5.0)	8.0 (2.2)	6.0 (3.0)	− 1.0 (3.3)	*Z* = − 3.53, *p <* .001
Worst pain (0–10)	8.0 (2.0)	4.0 (3.0)	− 3.0 (4.0)	8.0 (2.0)	7.0 (2.0)	− 1.0 (2.0)	*Z* = − 4.63, *p* < .001
Function (1–5)	4.0 (1.0)	3.0 (2.0)	−1.0 (2.0)	4.0 (1.2)	3.5 (1.0)	−0.5 (1.0)	*Z* = − 3.17, *p* = .002
Symptom-specific well-being (1–5)	5.0 (0.0)	4.0 (3.0)	− 1.0 (3.0)	5.0 (0.0)	5.0 (1.0)	0.0 (1.0)	*Z* = − 3.51, *p* < .001
Quality of life (1–5)	4.0 (2.0)	3.0 (1.0)	− 1.0 (2.0)	4.0 (1.3)	3.5 (1.0)	0.0 (1.0)	*Z* = − 4.59, *p* < .001
Social disability (1–5)	4.0 (2.0)	2.0 (3.0)	− 1.0 (2.0)	4.0 (2.0)	4.0 (2.0)	0.0 (1.3)	*Z* = − 2.87, *p =* .004
Work disability (1–5)	2.0 (4.0)	1.0 (3.0)	0.0 (1.0)	3.0 (4.0)	3.0 (4.0)	0.0 (2.0)	*Z* = −1.23, *p =* .218
Overall COMI score (0–10)	7.9 (2.6)	4.4 (3.6)	−2.6 (3.3)	7.8 (2.2)	6.9 (2.3)	−1.0 (2.0)	*Z* = − 4.66, *p* < .001

*COMI* Core Outcome Measures Index; *IQR* Interquartile range

The median change in individual COMI item scores, the worst pain score as well as the overall COMI score was significantly greater in patients who perceived the therapeutic intervention as helpful (“good outcome group”), compared to those who perceived it as less helpful (“poor outcome group”). The only exception was for the work disability item (*p* = 0.218).

### Test-retest reliability (stability)

There were relatively minor differences between the test and retest median scores (Table 5). Specifically, for four domains (leg pain, function, quality of life and social disability), we observed no changes in the median score. There were small changes in the overall COMI score (− 0.1) as well as the “back pain” item and the worst pain score (+ 0.5), and slightly more pronounced changes for the “symptom-specific well-being” item (− 1.0) as well as the “work disability” item (− 1.0). The ICCs ranged from 0.69 to 0.86, indicating good to excellent test-retest reliability. The highest ICC was found for the overall COMI score, while the lowest value was found for the “work disability” item.

**Table 5:** Test-retest reliability for each COMI domain and for the overall COMI score

	Median for test (*IQR*)	Median for retest (*IQR*)	ICC (95% CI)	SEM	MDC95%
Back pain (0–10)	3.5 (4.0)	4.0 (4.0)	0.80 [0.68; 0.87]	1.09	3.0
Leg pain (0–10)	3.0 (5.0)	3.0 (5.0)	0.71 [0.55; 0.81]	1.54	4.3
Worst pain (0–10)	4.5 (4.0)	5.0 (4.0)	0.83 [0.74; 0.89]	1.04	2.9
Function (1–5)	3.0 (2.0)	3.0 (2.0)	0.75 [0.61; 0.84]	0.51	1.4
Symptom-specific well-being (1–5)	4.0 (2.3)	3.0 (2.0)	0.79 [0.68; 0.87]	0.60	1.7
Quality of life (1–5)	3.0 (1.0)	3.0 (2.0)	0.76 [0.63; 0.84]	0.43	1.2
Social disability (1–5)	2.0 (3.0)	2.0 (3.0)	0.71 [0.55; 0.81]	0.79	2.2
Work disability (1–5)	2.0 (3.0)	1.0 (2.0)	0.69 [0.62; 0.80]	0.94	2.6
Overall COMI score (0–10)	5.0 (3.6)	4.9 (4.0)	0.86 [0.78; 0.91]	0.86	2.4

*COMI* Core Outcome Measures Index; *IQR* Interquartile range; *ICC* Intraclass correlation coefficients; *SEM* Standard errors of measurement, MDC: Minimum detectable change

Table 5 also contains data regarding the standard error of measurement (SEM) and minimum detectable change score (MDC95%). The SEM and MDC95% were largest for the “leg pain” item and smallest for the “quality of life” item, with SEM for the overall COMI score being 0.86 and MDC95% 2.4 points.

## Discussion

### Missing values, floor and ceiling effects

The response rate for COMI items was extremely good, with respondents in the general sample answering all of the individual COMI items. This finding is especially meaningful once we compare these results to those for the ODI. In the same sample of 353 individuals, a relatively high number of missing values (*N* = 124; 35.1%) was found for the “sex life” item of the ODI. This figure is slightly higher than that reported in previous studies; in the Polish COMI validation sample, for example, 23.0% did not answer the “sex life” question of the ODI. Many other COMI validation studies have also shown a very low number of missing values [7, 18], implying that the COMI items in general as well as in the Slovene version do not ask about information that could be considered as too sensitive (and thus not answered) by the respondent.

While most of the COMI items did not exhibit any meaningful ceiling effects (they were all in the 0–20% ideal range), the “work disability” item did show a slightly higher proportion of patients reporting the best status, although this was still far below the threshold that could be considered as adverse [15]. Interestingly, the same item has exceeded the ideal range of 0–20% in many previous COMI validations [7, 14, 21, 22] with some of these studies also showing a greater ceiling effect for the “social disability” item, although this was not observed in our sample. In contrast, the floor effects in the present study were somewhat more prominent and affected five items, namely: “quality of life”, “work disability”, “function”, “social disability”, and, in particular, “symptom-specific well-being”, with values for the latter exceeding 70% - the value considered adverse [15]. As with ceiling effects, this finding was not unexpected; a similar floor effect for the “symptom-specific well-being” item was also noted in many other COMI validation studies [7, 14, 21, 22] and could be attributed to the fact that the data were those of presurgical patients, who were generally in severe pain and not satisfied “to spend the rest of their life with their current symptoms”. Overall, the findings for floor and ceiling effects are in line with those reported in other COMI validation studies, which further serves to support the validity of the Slovene adaptation of the instrument.

### Construct validity

To assess the construct validity, the relationships between individual COMI items, the overall COMI score, and an already established questionnaire validated in the Slovene language, the Oswestry Disability Index, were analyzed. To confirm the hypothesis that the instruments measure a similar construct, the Spearman Rho should fall somewhere within the .40–.80 range [23]. In the present study, the overall COMI score correlated very well with the ODI (ρ = .76), demonstrating a relationship that was similar to, but slightly more pronounced than, that reported for the culturally relatively similar Polish version [7] as well as the Brazilian-Portuguese version [14]. The correlations with individual COMI items were also satisfactory; one item (“work disability”) showed a fair correlation with the ODI, three items (“symptom-specific well-being”, “leg pain”, and “social disability”) demonstrated good correlations with the ODI, and the remaining three items (“back pain”, “quality of life”, and “function”) showed correlations with the ODI that can be described as very good. These findings were largely in line with our expectations since previous studies [7] have also observed fair to good correlations between the individual COMI items and the overall ODI score. Overall, these results display a satisfactory construct validity of the Slovene version of the COMI low back questionnaire.

However, future studies should expand on construct validity analyses reported in the present paper by examining the relationship between the Slovene COMI and other comparison instruments besides the ODI, such as the Roland Morris disability questionnaire (RMQ) [24]. Additionally, future studies could further advance our understanding of the Slovene COMI validity by examining cross-cultural validity/measurement invariance (i.e., the degree to which the performance of the items on a translated measure are an adequate reflection of the original version).

### Responsiveness

The responsiveness analysis demonstrated that surgical patients from our sample showed a significant improvement in the median scores of individual COMI items as well as the overall COMI score, approximately 3 months after surgery. While our study is one of the few in the COMI literature that analyzed responsiveness, our result is very much in line with previous validation studies that did perform such analyses [8, 9, 18]. In the Hungarian validation study, for example, the effect size, pertaining to the change in mean scores 6 months after surgery, was large [18]. The analyses conducted on our sample with a different time frame (3 months) yielded a large effect size as well, showcasing the ability of the Core Outcome Measures Index to detect clinically important changes over time, even when the time period is relatively short.

Furthermore, the additional analyses also revealed that changes in COMI items and the overall COMI score differed between patients who rated the operation as being very helpful/helpful and those who perceived it as less helpful. Specifically, the COMI successfully captured a more pronounced improvement among “good outcome” patients, compared to “poor outcome” patients in all items except one (work disability remained relatively stable in both groups). While these results do offer valuable insight into the responsiveness characteristic of the Slovene COMI, future studies should investigate this further by determining the area under the curve of the Slovenian COMI.

### Test-retest reliability (stability)

The test-retest reliability of the overall COMI score (Slovene version) was found to be excellent despite the fact that the time lag in between the two COMI applications was significantly longer than in some previous COMI validations. Our findings thus represent an important contribution to the existing literature, supporting Mannion and colleagues [9] who found a significant reduction in COMI scores from pre-surgery to 3-months post-surgery, with the values then remaining stable up to 2 years after surgery. In our case, we have also observed a significant reduction in COMI scores post-surgery (the responsiveness part) and found that these values remained relatively unchanged approximately half a year after the therapeutic intervention (the reliability part [25];).

Lastly, the minimum detectable change of the Slovene COMI total score (2.4) was slightly higher than that published for the Hungarian (1.6 [18];), Brazilian-Portuguese (1.7 [14];), and Polish version (1.8 [7];), but relatively similar to the French (2.0 [21];) and Norwegian version (2.2 [21];). As such, the MDC for the sum scale is only marginally poorer than in some former studies and within range of that reported for other low back pain outcome instruments [26, 27]. The Slovene version of COMI thus exhibits acceptable minimum detectable change, meaning that a change of more than 2.4 points at the COMI index needs to be observed to be labeled as a real change (and not the measurement error). However, as the MDC is largely dependent on the ICC and standard deviation (*SD)*, future studies should investigate test-retest reliability and MDC on a sample of stable patients who fill out the questionnaire approximately 1–2 weeks apart instead of a longer time period as used in the present study.

## Conclusion

The Slovene version of COMI is a valid, cross-culturally adapted and reliable instrument for use in Slovene-speaking patients. Its availability in Slovene should encourage research and the publication of clinical studies in patient with low back problems in Slovenia.

## Data Availability

The datasets during and/or analyzed during the current study are available from the corresponding author on reasonable request.

## Abbreviations

COMI: Core Outcome Measures Index
ODI: Oswestry Disability Index
LBP: Low back pain
ICC: Intraclass correlation coefficients
SEM: Standard errors of measurement
IQR: Interquartile range
MDC: Minimum detectable change
SD: Standard deviation
